# Incidence and predictors of mortality among preterm neonates admitted to the neonatal intensive care unit of Wallaga University Comprehensive Specialized Hospital, Nekemte Town, East Wallaga, Oromia, Ethiopia: a retrospective cohort study

**DOI:** 10.1186/s12887-026-07059-z

**Published:** 2026-06-03

**Authors:** Minase Shenkute, Fikadu Enkosa, Emiru Merdassa

**Affiliations:** 1 Department of Pediatrics and Child Health, School of Medicine, Institute of Health Science, Dilla University, Dilla Town, Ethiopia; 2 Department of Pediatrics and Child Health, School of Medicine, Institute of Health Science, Wallaga University, Nekemte Town, Ethiopia; 3 Department of Public Health, Institute of Health Science, Wallaga University, Nekemte Town, Ethiopia

**Keywords:** NICU, WUCSH, Incidence of mortality, Predictors of mortality Preterm

## Abstract

**Background:**

Preterm neonatal mortality remains a major public health challenge worldwide. Despite the availability of modifiable risk factors and cost effective interventions, data on incidence of mortality of preterm neonates in the study area is lacking. Generating local data is essential to inform tailored interventions, strengthen neonatal care services, and support national efforts to reduce neonatal mortality.

**Objective:**

To assess incidence and predictors of mortality among preterm neonates admitted to the neonatal intensive care unit of Wallaga University Comprehensive Specialized Hospital, Nekemte Town, Oromia, Ethiopia, from July 1, 2022, to June 30, 2024.

**Methods:**

An institution-based retrospective cohort study was conducted among 264 preterm neonates admitted to the NICU from July 1, 2022 to June 30, 2024 and study subjects were selected using systematic random sampling technique. Data were collected using a structured checklist, key variables including ANC follow up, place of delivery, gestational age, and birth weight were extracted from medical records. Data entered via EpiData version 4.6, and subsequently analyzed using STATA version 14.0. The Cox proportional hazards model assumption was checked. A bivariable Cox regression analysis was fitted and those variable with *p* < 0.2 were included in the multivariable analysis. Finally, statistical significance was declared at a *p*-value < 0.05.

**Results:**

Of the 259 preterm neonates included in the study, 42 died during the follow-up period, with an incidence proportion of 16.2%. The median survival time was 28 days (IQR: 22–30), with a total follow-up of 2,737 neonate-days. The overall incidence rate of mortality was 15.3 per 1,000 neonate-days (95% CI: 11.3–20.7). In the multivariable Cox proportional hazards regression analysis, lack of ANC follow-up (AHR: 2.27; 95% CI: 1.13–4.57), home delivery (AHR: 7.74; 95% CI: 1.99–30.03), and neonatal hypothermia (AHR: 4.11; 95% CI: 1.55–10.85) were significantly associated with increased risk of mortality, while antenatal steroid use reduced the risk of death by 56% (AHR: 0.44; 95% CI: 0.21–0.92).

**Conclusion and recommendation:**

Lack of ANC follow-up, home delivery, and neonatal hypothermia were significant predictors of preterm neonatal mortality, while antenatal steroid use reduced the risk of death. Strengthening antenatal care utilization, promoting institutional delivery, improving thermal care, and ensuring appropriate antenatal corticosteroid administration may help improve survival among preterm neonates.

**Supplementary Information:**

The online version contains supplementary material available at https://doi.org/10.1186/s12887-026-07059-z.

## Background

Preterm birth, defined as delivery before 37 completed weeks of gestation, remains a major global public health problem and a leading cause of neonatal morbidity and mortality. Based on gestational age, preterm birth is categorized as moderate to late preterm (32–37 weeks), very preterm (28– < 32 weeks), and extremely preterm (< 28 weeks). Globally, nearly 1 in 10 newborns is born preterm, with the greatest burden occurring in Southern Asia and sub-Saharan Africa, where access to quality neonatal care remains limited and neonatal survival is comparatively low [1]. The prevalence of preterm birth ranges from 5 to 18% across different countries and settings [2, 3]. In Ethiopia, a meta-analysis reported a pooled prevalence of preterm birth of 11.4%, indicating that preterm birth remains a significant maternal and child health challenge [8].

Preterm birth may occur spontaneously or be medically indicated due to maternal or fetal complications. Several maternal, obstetric, and neonatal factors contribute to poor outcomes among preterm neonates. Previous studies conducted in Ethiopia and other sub-Saharan African countries have identified factors such as pregnancy-induced hypertension, premature rupture of membranes, maternal anemia, multiple gestation, low birth weight, birth asphyxia, neonatal sepsis, respiratory distress syndrome (RDS), and absence of kangaroo mother care (KMC) as major contributors to preterm neonatal mortality [6–8, 13]. These conditions increase vulnerability to severe complications during the neonatal period and contribute substantially to neonatal deaths in resource-limited settings.

Preterm neonates are highly susceptible to both immediate and long-term complications due to organ immaturity. Common early complications include respiratory distress syndrome, neonatal sepsis, hypothermia, intraventricular hemorrhage, necrotizing enterocolitis, and feeding difficulties [9, 10]. These complications are major causes of mortality among preterm neonates, particularly in low-income countries where advanced neonatal intensive care services are limited. Although studies from high-income countries demonstrate improved survival among extremely preterm neonates due to advanced neonatal care and technology, such comparisons highlight the persistent disparities in neonatal outcomes between resource-rich and resource-limited settings [1, 12]. In sub-Saharan Africa, mortality among extremely preterm neonates remains disproportionately high because of shortages of skilled healthcare providers, inadequate neonatal equipment, and limited access to specialized neonatal care.

Globally, complications related to preterm birth account for nearly one million deaths annually and contribute substantially to neonatal and under-five mortality [4]. In Ethiopia, preterm neonatal mortality remains a major public health concern despite ongoing efforts to strengthen neonatal care services. Studies conducted in different parts of the country have reported high mortality rates among preterm neonates, with predictors including gestational age below 32 weeks, low Apgar score, low birth weight, respiratory distress syndrome, neonatal sepsis, jaundice, birth asphyxia, and lack of kangaroo mother care [13].

Several evidence-based interventions, including antenatal corticosteroid administration, neonatal resuscitation, kangaroo mother care, delayed cord clamping, infection prevention, and early initiation of breastfeeding, have been shown to improve survival among preterm neonates [1, 14, 15]. Ethiopia has incorporated many of these interventions into its National Neonatal and Child Survival Strategy to reduce neonatal mortality. However, implementation challenges such as shortages of trained professionals, inadequate medical supplies, and limited neonatal intensive care capacity continue to affect the quality of neonatal care services, especially in resource-limited settings [13, 16].

Although studies on preterm neonatal mortality have been conducted in some parts of Ethiopia, there is limited evidence regarding the incidence and predictors of mortality among preterm neonates in the study area. Generating local evidence on the incidence and predictors of mortality among preterm neonates is essential to support context-specific interventions, improve neonatal care practices, and strengthen efforts aimed at reducing neonatal mortality in Ethiopia.

## Methods and materials

### Study design

An institution-based retrospective cohort study was conducted. It involved review of charts of preterm neonates admitted between 1 July, 2022 and 30 June, 2024 GC.

### Study setting

This study was carried out at WUCSH located in Nekemte town, the capital city of East Wallaga, lies 331 km west of Addis Ababa. WUCSH, a teaching and specialized hospital, serves an estimated population of 5 million within its catchment area. The hospital offers NICU services, to both inborn and out born term and preterm neonates.

### Population

#### Population

All preterm neonates admitted to the neonatal intensive care unit of WUCSH.

#### Study population

All preterm neonates admitted to the neonatal intensive care unit of WUCSH from July 1, 2022 to June 30, 2024 GC.

#### Study unit

Each randomly selected preterm neonate's chart from the HMIS registry book.

### Eligibility criteria

#### Inclusion criteria

All medical charts of preterm neonates admitted to the NICU from July 1, 2022 to June 30, 2024 GC were eligible for inclusion in the study.

#### Exclusion criteria

Medical charts of preterm neonates with incomplete key information, including discharge outcome, date of admission, date of discharge, and other essential baseline variables, were excluded from the study.

### Sample size determination

The sample size was determined using the Cox proportional hazards model in STATA version 14.0 based on predictors identified from a previous study conducted in Southern Ethiopia. The STATA command used for sample size calculation was:

power cox, hratio(2.3) failprob(0.255) wdprob(0.15).

The assumptions used were 95% confidence level, 80% statistical power, expected proportion of mortality (failure probability) of 25.5%, and withdrawal probability of 15% to compensate for incomplete records. Hazard ratios from previously identified predictors including lack of ANC, primiparity, pregnancy complications, resuscitation at birth, and absence of KMC were considered [17] (Table 1).

**Table 1:** Sample size determination for incidence and predictors of preterm mortality in the NICU at WUCSH, Nekemte Town, East Wallaga, Oromia, Ethiopia from July 1, 2022 to June 30, 2024 GC

No	Predictor variables	HR	Power (%)	Total sample size	References
1	lack of ANC	7.1	80	38	[17]
2	Primiparity	2.3	80	209	[17]
3	Pregnancy complications	3.4	80	97	[17]
4	Resuscitation at birth	2.1	80	**264**	[17]
5	Absence of KMC	9.3	80	30	[17]

The largest calculated sample size (264) was selected as the final sample size

### Sampling techniques

Study subjects were selected using a systematic random sampling (SRS) technique. The sampling interval (K) was determined by dividing the total number of preterm neonates over the study period by the required sample size (K = N/n). During the study period, a total of 569 preterm neonates were admitted to the NICU and constituted the study population (*N* = 569). The sampling interval: $k=\frac{N}{n}=\frac{569}{264}=2.1$

The sampling frame consisted of patient records listed in the HMIS registry book, and every 2nd record was selected until the total sample size was reached. The first record was chosen randomly using a lottery method among preterm neonates admitted on the first day of the study period (July 1, 2022). If a selected chart was missing, the next available record in sequence was used.

### Study variables

#### Dependent variable

Incidence of mortality.

#### Independent variables

##### Socio-demographic variables

Maternal age, residence, and gender of the baby.

##### Maternal and obstetrics variables

Parity, ANC follow-up, antenatal steroid use, pregnancy induced hypertension, GDM, preterm labor, maternal infection (chorioamnionitis), place of delivery, and mode of delivery.

##### Neonatal and clinical variables

Gestational age, birth weight, APGAR score, resuscitation at birth, and hospital complications.

### Operational definition

#### Censored

Defined when preterm neonates discharge improved, lost to follow up or referred to other institution within the study period [18].

#### Event

Referred to the death of preterm neonates during their hospital stay [18].

#### Follow up time

The time starting from admission until either an event or censorship occurs, with a maximum follow-up period of 30 days [18].

#### Time scale

Days from the admission of a preterm neonate [18].

#### Preterm

Neonates born alive before 37 weeks of pregnancy are completed [1].

#### Moderate to late preterm

Infants are those born between 32 and 37 weeks of gestation [1].

#### Very preterm

Is defined as 28 weeks of gestation to fewer than 32 weeks of gestation [1].

#### Extremely preterm

Is designated less than 28 weeks of gestational age [1].

#### Respiratory distress

Clinically diagnosed in premature infants with rapid, labored breathing, often with grunting, flaring nostrils, and chest wall retractions [19].

#### Hypothermia

Defined as skin (axillary) temperature less than 36.5^0^C [19].

#### Apnea

Defined as absence of air flow for ≥ 20 s or less than that if it is accompanied by bradycardia (heart rate < 100/min) or cyanosis [19].

#### Necrotizing enterocolitis

A clinical diagnosis can be established when a neonate presents with symptoms such as abdominal distention, feeding intolerance, vomiting, bloody stools, loose stools, abdominal wall erythema, and vital sign instability [19].

#### Sepsis

Clinical diagnosis is based on the presence of symptoms such as temperature instability, respiratory distress, feeding difficulties, lethargy, seizures, and jaundice in neonates with known risk factors, including maternal infection, PROM, or the need for resuscitation at birth. Further divided into Early onset (below 7 days) and Late onset (7–28 days) sepsis [19].

#### First-line antibiotics

Refer to the initial antibiotics administered, commonly including ampicillin and gentamicin [19].

#### Second-line antibiotics

Are those that are either added or changed into if there is no improvement after 48 h or if the infant’s condition worsens [19].

### Data collection tools and procedures

#### Before data collection

The list of preterm neonates admitted to the NICU during study period was obtained from the HMIS registry book. Then, the required sample size was selected using a systematic random sampling technique based on the calculated sampling interval.

#### During data collection

Data were extracted retrospectively from the selected medical records using a standardized structured checklist adapted from previous related studies. Information collected included socio-demographic variables (maternal age, residence, and sex of neonate), maternal and obstetric variables (ANC follow-up, parity, antenatal steroid use, pregnancy-induced hypertension, gestational diabetes mellitus, preterm labor, maternal infection, place of delivery, and mode of delivery), and neonatal and clinical-related variables (APGAR score, resuscitation at birth, and hospital complications) [8, 13, 22, 25–32]. Birth weight and gestational age at birth were classified according to WHO standards [1]. Data was collected by trained neonatal nurses from September 15 to October 15, 2024 GC.

#### After data collection

The extracted data were checked daily for completeness, consistency, and accuracy by the principal investigator before data entry and analysis.

### Data processing, management, and analysis

The data were coded, cleaned, and entered into EpiData version 4.6 and then exported to STATA version 14 for analysis.

Descriptive statistics such as frequencies, percentages, median, and interquartile range were used to summarize the data and presented using tables, graphs, and texts.

Multicollinearity test was done, and no significant correlation was detected among the independent variables.

The Incidence proportion and Incidence Density Rate (IDR) was calculated for the entire study period. Subsequently, the number of mortalities within the follow-up period was divided by the total neonate-time at risk during follow-up and reported per 1000-neonate days. The Kaplan–Meier curve was used to estimate median survival time, and log-rank tests was used to compare survival curves.

The Cox proportional hazards assumption was also checked using both graphs and Schoenfeld’s residual test, with variables having p-values greater than 0.05 being considered as fulfilling the assumption. The Cox-Snell residuals were also used to assess the overall goodness-of-fit of the Cox proportional hazards model. Bivariable cox regression was first fitted and those independent variables having *p*-value < 0.2 were included in the multivariable analysis. Finally, statistical significance was declared at a *p*-value < 0.05. Hazard Ratios (HR) with 95% Confidence Intervals (CI) was used to assess the relationship between factors associated with the occurrence of death.

Variables with zero events during the follow-up period (UTI, Intrauterine infection, APH, Hepatitis B infection, and first-line antibiotics) were excluded from Cox regression due to inability to estimate hazard ratios, but were included in descriptive statistics.

Maternal age was initially recorded in 15–19, 20–34 & ≥ 35 years age bands, but was re-grouped into < 20 & ≥ 20 years because the ≥ 35 years group had zero deaths. Similarly, Place of ANC Follow up was re-categorized into Health center & Hospital due to zero events occurs in Tertiary Hospitals.

### Ethical consideration

Ethical clearance was obtained from the Wallaga University Institute of Health Science Research Ethics Committee with reference number IHSREC/005/041/2024, dated December 25, 2024. Then, Clinical director’s office wrote an official letter of permission to NICU and Record room head for securing permission. After obtaining permission from the concerned body, data gathered from charts. Information obtained from the charts was kept confidentially by not recording neonates and their mother’s name on check lists.

## Results

### Description of study participants

**Fig. 1:**
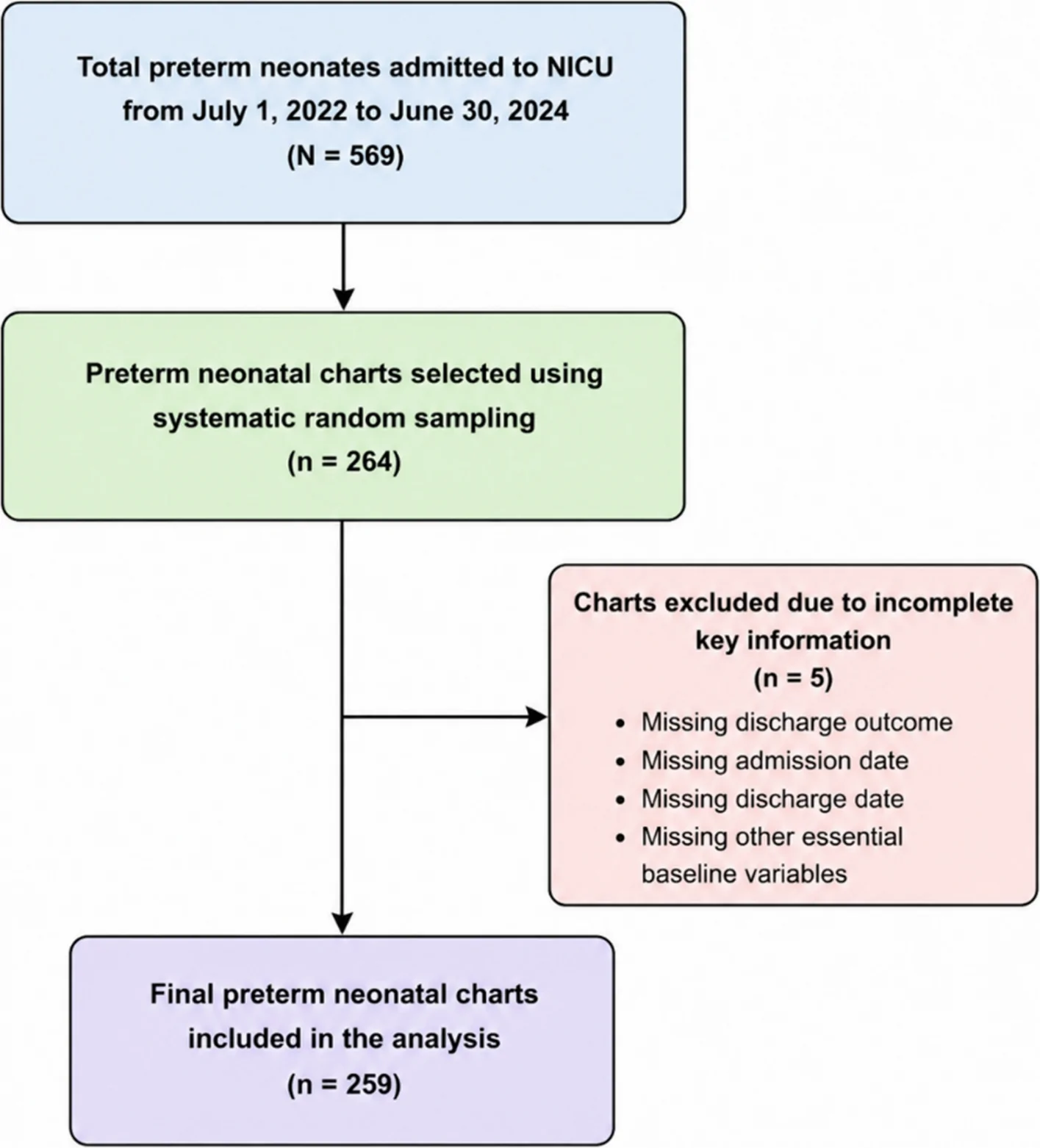
Flow diagram showing selection of preterm neonatal charts included in the study conducted at WUCSH, Nekemte Town, East Wallaga, Oromia, Ethiopia, from July 1, 2022 to June 30, 2024 GC

During the study period, a total of 569 preterm neonatal admissions were recorded. From these, 264 medical charts were selected using systematic random sampling. During data extraction, 5 charts were excluded because of incomplete key information such as discharge outcome, date of admission, date of discharge, and other essential baseline data. Finally, 259 preterm neonatal charts were included in the final analysis (Fig. 1).

### Socio-demographic characteristics and antenatal care status of mothers

According to this study, the mothers' ages ranged from 15 to 38 years, with a mean age of 24.4 ± 1.4 years. The most common age group was ≥ 20 years (88.8%). The majority of the mothers had one or two children 210(81.1%) and lived in rural areas 176(67.9%).

The majority of mothers had antenatal care (ANC) follow-up, with 192 (74.1%) attending, of whom 120 (62.5%) visited a health center. Additionally, 180 (93.7%) of them had fewer than four ANC visits, which is below the current national guideline recommendation of at least eight visits. During pregnancy, 116 (44.8%) experienced pregnancy-related complications. The most common complications were pregnancy-related hypertension (30.1%), premature rupture of membranes (PROM) (6.6%), and malaria (6.2%), as shown in Table 2 below.

**Table 2:** Socio-demographic characteristics of mothers of preterm neonates at WUCSH, Nekemte Town, East Wallaga, Oromia, Ethiopia, from July 1, 2022 to June 30, 2024 GC

Variables	Variables Frequency (%)	Survival Status		
		**Event (%)**		**Censored (%)**
Age of the mother	< 20yrs	29(11.2)	3(1.2)	26(10)
	≥ 20yrs	230(88.8)	39(15.1)	191(73.7)
Residence	Urban	83(32.1)	8(3.1)	75(28.9)
	Rural	176(67.9)	34(13.1)	142(54.8)
Parity	1-2birth	210(81.1)	37(14.3)	173(66.8)
	3-5birth	40(15.4)	4(1.5)	36(13.9)
	> 5birth	9(3.5)	1(0.4)	8(3.1)
ANC Follow up	Yes	192(74.1)	15(5.8)	177(68.3)
	No	67(25.8)	27(10.4)	40(15.4)
Place of ANC Follow up	Health Center	120(62.5)	13(6.8)	107(55.7)
	Hospital	72(37.5)	2(1.1)	70(36.4)
Frequency of ANC Follow up	< 4 times	180(93.7)	14(7.3)	166(86.4)
	≥ 4 times	12(6.3)	1(0.5)	11(5.8)
Antenatal Steroid use	Yes	132(50.9)	15(5.8)	117(45.1)
	No	127(49.1)	27(10.5)	100(38.6)
Pregnancy related complication	Yes	116(44.8)	26(10)	90(34.8)
	No	143(55.2)	16(6.2)	127(49)
Pregnancy induced Hypertension	Yes	78(30.1)	16(6.2)	62(23.9)
	No	181(69.9)	26(10)	155(59.9)
PROM	Yes	17(6.6)	4(1.5)	13(5.1)
	No	242(93.4)	38(14.7)	204(78.7)
UTI	Yes	3(1.2)	0(0)	3(1.2)
	No	256(98.8)	42(16.2)	214(82.6)
Intrauterine infection (Chorioamnionitis)	Yes	2(0.8)	0(0)	2(0.8)
	No	257(99.2)	42(16.2)	215(83)
Malaria	Yes	16(6.2)	8(3.1)	8(3.1)
	No	243(93.8)	34(13.1)	209(80.7)
APH	Yes	2(0.8)	0(0)	2(0.8)
	No	257(99.2)	42(16.2)	215(83)
Hepatitis B virus status	Positive	4(1.5)	0(0)	4(1.5)
	Negative	255(98.5)	42(16.2)	213(82.3)

### Profile of preterm neonates

**Fig. 2:**
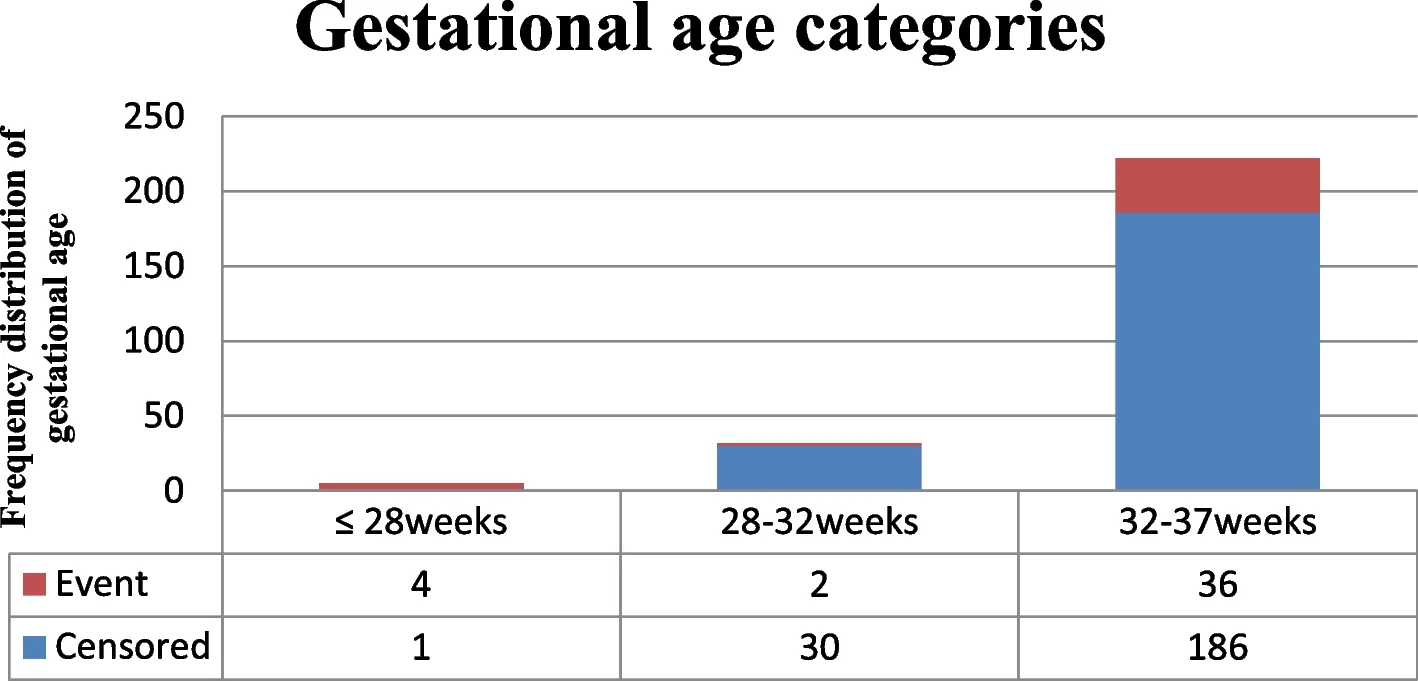
Frequency distribution of gestational age categories of preterm neonates at WUCSH, Nekemte Town, East Wallaga, Oromia, Ethiopia, from July 1, 2022 to June 30, 2024 GC

According to this study, most of the preterm neonates were between 32 to 37 weeks 222(85.7%), with 5 (1.9%) being extremely preterm, as shown in Fig. 2 below.

The male preterm neonates accounts for 183 (70.7%) of the total cases. Majority of preterm neonates had a birth weight ranging from 1500 to 2400 g, with 181 (69.9%). Additionally, most of the preterm neonates were born in hospital, comprising 229 (88.4%) of the total cases, as shown in Table 3 below.

**Table 3:** Profile of preterm neonates at WUCSH, Nekemte Town, East Wallaga, Oromia, Ethiopia, from July 1, 2022 to June 30, 2024 GC

Variables		Variables Frequency (%)	Survival Status	
			**Event (%)**	**Censored (%)**
Sex of the neonate	Male	183(70.7)	29(11.2)	154(59.5)
	Female	76(29.3)	13(5.0)	63(24.3)
Place of birth	Home delivery	6(2.3)	3(1.16)	3(1.16)
	Health Center	24(9.3)	6(2.3)	18(7)
	Hospital	229(88.4)	33(12.7)	196(75.7)
Gestational age	≤ 28 weeks	5(1.9)	4(1.5)	1(0.4)
	28–32 weeks	32(12.4)	2(0.8)	30(11.6)
	32–37 weeks	222(85.7)	36(13.9)	186(71.8)
Birth weight	< 1000gm	5(1.9)	4(1.5)	1(0.4)
	1000-1499gm	73(28.2)	10(3.9)	63(24.3)
	1500-2400gm	181(69.9)	28(10.8)	153(59.1)
APGAR score	Known	224(86.5)	35(13.5)	189(73)
	Unknown	35(13.5)	7(2.7)	28(10.8)
Fifth minute APGAR score	≥ 7	196(87.5)	20(8.9)	176(78.6)
	< 7	28(12.5)	15(6.7)	13(5.8)
Resuscitation at birth	Yes	44(16.9)	21(8.1)	23(8.8)
	No	215(83.1)	21(8.1)	194(75)

### Preterm complications

The majority of preterm neonates, 254 (98.1%), experienced one or more hospital complications. The most common complications were hypothermia in 179(69.1%) of the cases, followed by RDS in 119 (45.9%), EONS in 105(40.5%), jaundice in 54(20.8%), and Apnea in 23(8.8%), as shown in Table 4 below.

**Table 4:** Preterm complications at WUCSH, Nekemte Town, East Wallaga, Oromia, Ethiopia, from July 1, 2022 to June 30, 2024 GC

Variables		Variables Frequency (%)	Survival Status	
			**Event (%)**	**Censored (%)**
Preterm complications	Yes	254(98.1)	42(16.2)	212(81.9)
	No	5(1.9)	0(0)	5(1.9)
Hypothermia	Yes	179(69.1)	35(13.5)	144(55.6)
	No	80(30.9)	7(2.7)	73(28.2)
RDS	Yes	119(45.9)	29(11.2)	90(34.7)
	No	140(54.1)	13(5.0)	127(49.1)
EONS	Yes	105(40.5)	21(8.1)	84(32.4)
	No	154(59.5)	21(8.1)	133(51.4)
Jaundice	Yes	54(20.8)	18(6.9)	36(13.9)
	No	205(79.2)	24(9.3)	181(69.9)
Apnea	Yes	23(8.8)	7(2.7)	16(6.1)
	No	236(91.1)	35(13.5)	201(77.6)
Seizure	Yes	16(6.2)	6(2.3)	10(3.9)
	No	243(93.8)	36(13.9)	207(79.9)
NEC	Yes	14(5.4)	8(3.1)	6(2.3)
	No	245(94.6)	34(13.1)	211(81.5)
IVH	Yes	13(5.1)	2(0.8)	11(4.3)
	No	246(94.9)	40(15.4)	206(79.5)
Anemia	Yes	12(4.6)	7(2.7)	5(1.9)
	No	247(95.4)	35(13.5)	212(81.9)
Congenital Malformation	Yes	6(2.3)	2(0.8)	4(1.5)
	No	253(97.7)	40(15.4)	213(82.3)

### Treatment given to preterm neonates

In this study, preterm neonates received a range of treatments including oxygen, antibiotics, aminophylline, phototherapy, phenobarbital, and blood transfusions. The three most commonly given treatments were first-line antibiotic therapy 198(76.4%), followed by oxygen therapy 145(55.9%) and second-line antibiotic therapy 83(41.9%), as shown in Table 5 below.

**Table 5:** Treatment given to preterm neonates at WUCSH, Nekemte Town, East Wallaga, Oromia, Ethiopia, from July 1, 2022 to June 30, 2024 GC

Variables		Variables Frequency (%)	Survival Status	
			**Event (%)**	**Censored (%)**
First-line antibiotics	Yes	198(76.4)	42(16.2)	156(60.2)
	No	61(23.6)	0(0)	61(23.6)
Oxygen therapy	Yes	145(55.9)	40(15.4)	105(40.5)
	No	114(44.1)	2(0.8)	112(43.3)
Second-line antibiotics	Yes	83(41.9)	26(13.1)	57(28.8)
	No	115(58.1)	16(8.1)	99(50)
Aminophylline	Yes	31(11.9)	15(5.8)	16(6.1)
	No	228(88.1)	27(10.4)	201(77.7)
Phototherapy	Yes	29(11.2)	5(1.9)	24(9.3)
	No	230(88.8)	37(14.3)	193(74.5)
Blood transfusion	Yes	13(5.1)	6(2.4)	7(2.7)
	No	246(94.9)	36(13.9)	210(81)
Phenobarbital	Yes	11(4.2)	6(2.3)	5(1.9)
	No	248(95.8)	36(13.9)	212(81.9)

### Outcome of preterm neonates

**Fig. 3:**
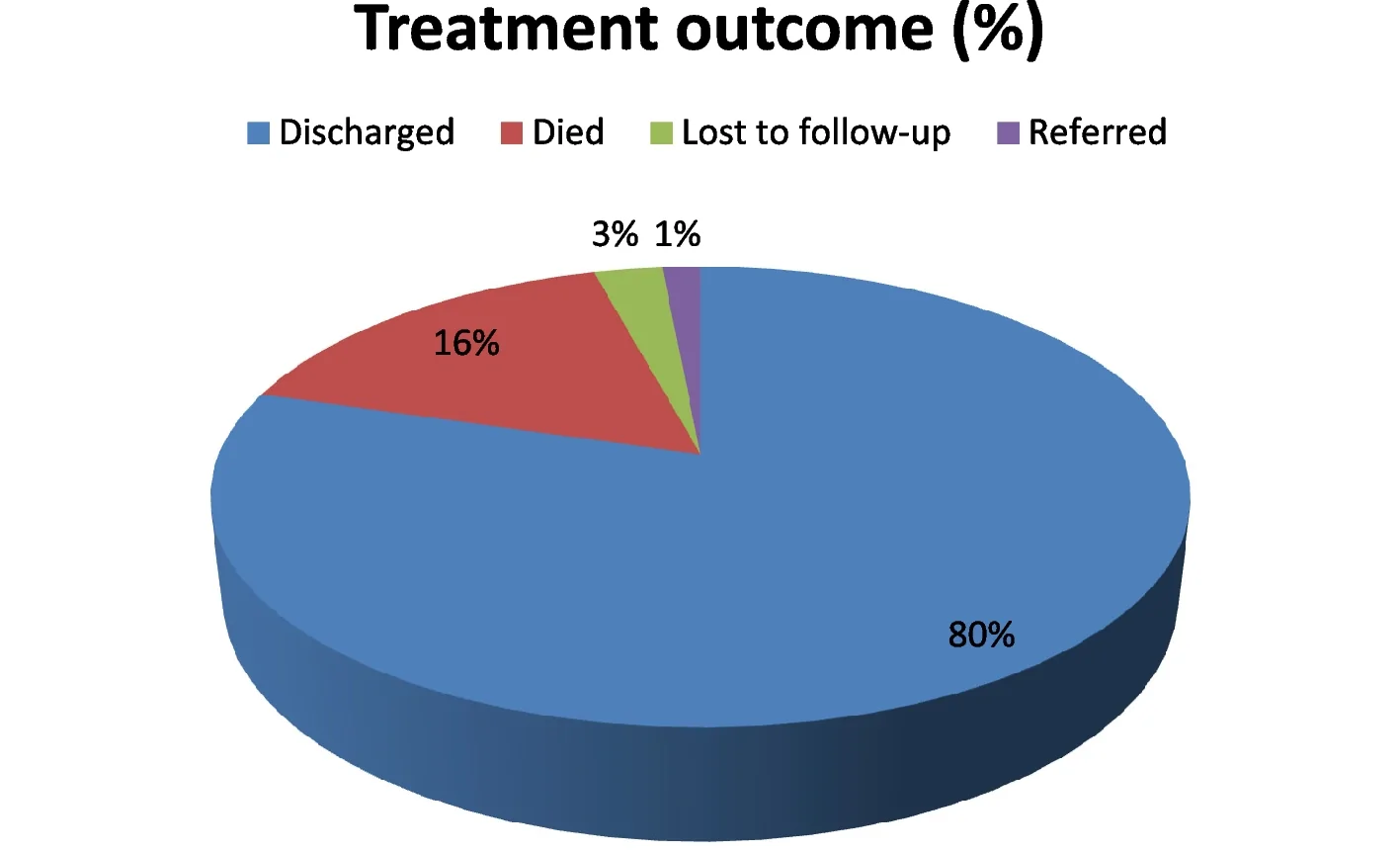
Outcomes of preterm neonates at WUCSH, Nekemte Town, East Wallaga, Oromia, Ethiopia, from July 1, 2022 to June 30, 2024 GC

Figure 3 shows the outcomes of 259 preterm neonates admitted to the NICU at WUCSH between July 1, 2022, and June 30, 2024 GC and included in the study. Of the neonates, 206 (79.5%) were discharged after clinical improvement, 42 (16.2%) died, 4 (1.5%) were referred, and 7 (2.7%) were lost to follow-up.

This figure shows valuable insight into the overall prognosis of preterm neonates during the study period. While the majority (over 79.5%) improved with treatment, the incidence proportion of 16.2% highlights the severity of the condition in this vulnerable population. The relatively high mortality underscores the critical need for improved preventing complication, optimized neonatal care, and early identification of risk factors associated with poor outcomes.

### Incidence of mortality among preterm neonates admitted to NICU

Out of 259 preterm neonates included in the study, 42 died during the follow-up period, makes an incidence proportion of 16.2%. The median survival time was 28 days, with an interquartile range (IQR) of 22 to 30 days. During follow-up time, a total of 2737 neonate-day observation were observed with a minimum of 1 day and maximum of 30 days follow-up time. The overall mortality incidence rate was 15.3 per 1,000 preterm neonates-day (CI: 11.3, 20.7).

### Over-all Kaplan- Meier failure estimate

**Fig. 4:**
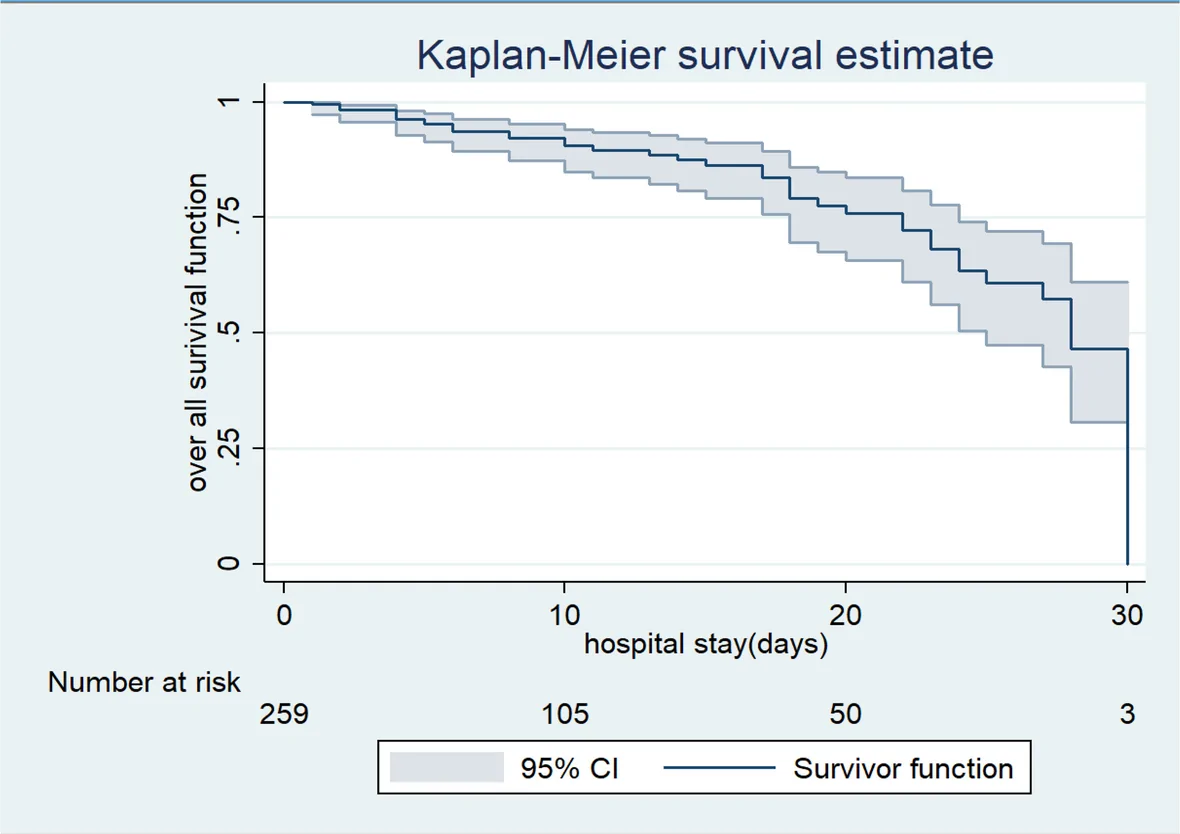
Overall Kaplan–Meier Survival probability curve of preterm neonates at WUCSH, Nekemte Town, East Wallaga, Oromia, Ethiopia, from July 1, 2022 to June 30, 2024 GC

The overall Kaplan–Meier estimate showed that the probability of survival of preterm neonates was higher on the first day of admission, with increased failure to survive throughout the follow-up period. Notably, sharper drops in survival were observed after around 15 days, as shown in Fig. 4 below.

### Predictors of Mortality among preterm neonates admitted NICU

The study identified several significant predictors of neonatal mortality among preterm neonates admitted to the NICU. Lack of antenatal care (ANC) follow-up was associated with a more than twofold increased risk of mortality (AHR: 2.27, 95% CI: 1.13–4.57). Neonates born at home were particularly vulnerable, with a markedly elevated mortality risk (AHR: 7.74, 95% CI: 1.99–30.03) compared to those born in hospital. Hypothermia is also strong independent predictors, with adjusted hazard ratios of 4.11 (95% CI: 1.55–10.85). Importantly, antenatal steroid use demonstrated a protective effect, reducing preterm mortality by 55.7% (AHR: 0.44, 95% CI: 0.21–0.92). These findings highlight the critical importance of quality antenatal care, including hospital delivery, proper thermal regulation, and interventions including antenatal steroid use, in improving preterm neonatal survival (Table 6).

**Table 6:** Results of the bivariate and multivariate Cox regression analysis of predictors of preterm mortality at WUCSH, Nekemte Town, East Wallaga, Oromia, Ethiopia, from July 1, 2022 to June 30, 2024 GC

Variables	Category	Status			CHR (95% CI)	AHR (95%CI)
		**Total (%)**	**Event (%)**	**Censored (%)**		
ANC Follow up	Yes	192(74.1)	15(5.8)	177(68.3)	1	1
	No	67(25.9)	27(10.5)	40(15.4)	3.42(1.80–6.51)	2.27(1.13–4.57) *
Antenatal Steroid use	Yes	132(50.9)	15(5.8)	117(45.1)	0.31(0.15–0.61)	0.44(0.21–0.92) *
	No	127(49.1)	27(10.5)	100(38.6)	1	1
Place of birth	Home delivery	6(2.3)	3(1.16)	3(1.16)	9.95(2.87–34.3)	7.74(1.99–30.03) **
	Health Center	24(9.3)	6(2.3)	18(7)	2.41(1.00–5.83)	1.65(0.61–4.45)
	Hospital	229(88.4)	33(12.7)	196(75.7)	1	1
Resuscitation at birth	Yes	44(16.9)	21(8.1)	23(8.8)	2.21(1.17–4.19)	1.84(0.86–3.89)
	No	215(83.1)	21(8.1)	194(75)	1	1
Hypothermia	Yes	179(69.1)	35(13.5)	144(55.6)	2.96(1.29–6.8)	4.11(1.55–10.85) **
	No	80(30.9)	7(2.7)	73(28.2)	1	1
Anemia	Yes	13(5.1)	8(3.1)	5(2)	2.32(1.01–5.31)	2.00(0.79–5.05)
	No	246(94.9)	34(13.1)	212(81.8)	1	1
IVH	Yes	13(5.1)	2(0.8)	11(4.3)	0.37(0.08–1.57)	0.67(0.14–2.99)
	No	246(94.9)	40(15.4)	206(79.5)	1	1
Seizure	Yes	16(6.2)	6(2.3)	10(3.9)	1.82(0.75–4.39)	2.20(0.78–6.23)
	No	243(93.8)	36(13.9)	207(79.9)	1	1

*AHR* Adjusted Hazard Ratio, *CHR* Crude Hazard Ratio

^*^ Statistically significant at *p* < 0.05; ** statistically significant at *p* < 0.01

### Kaplan–Meier curve for different categorical variable

**Fig. 5:**
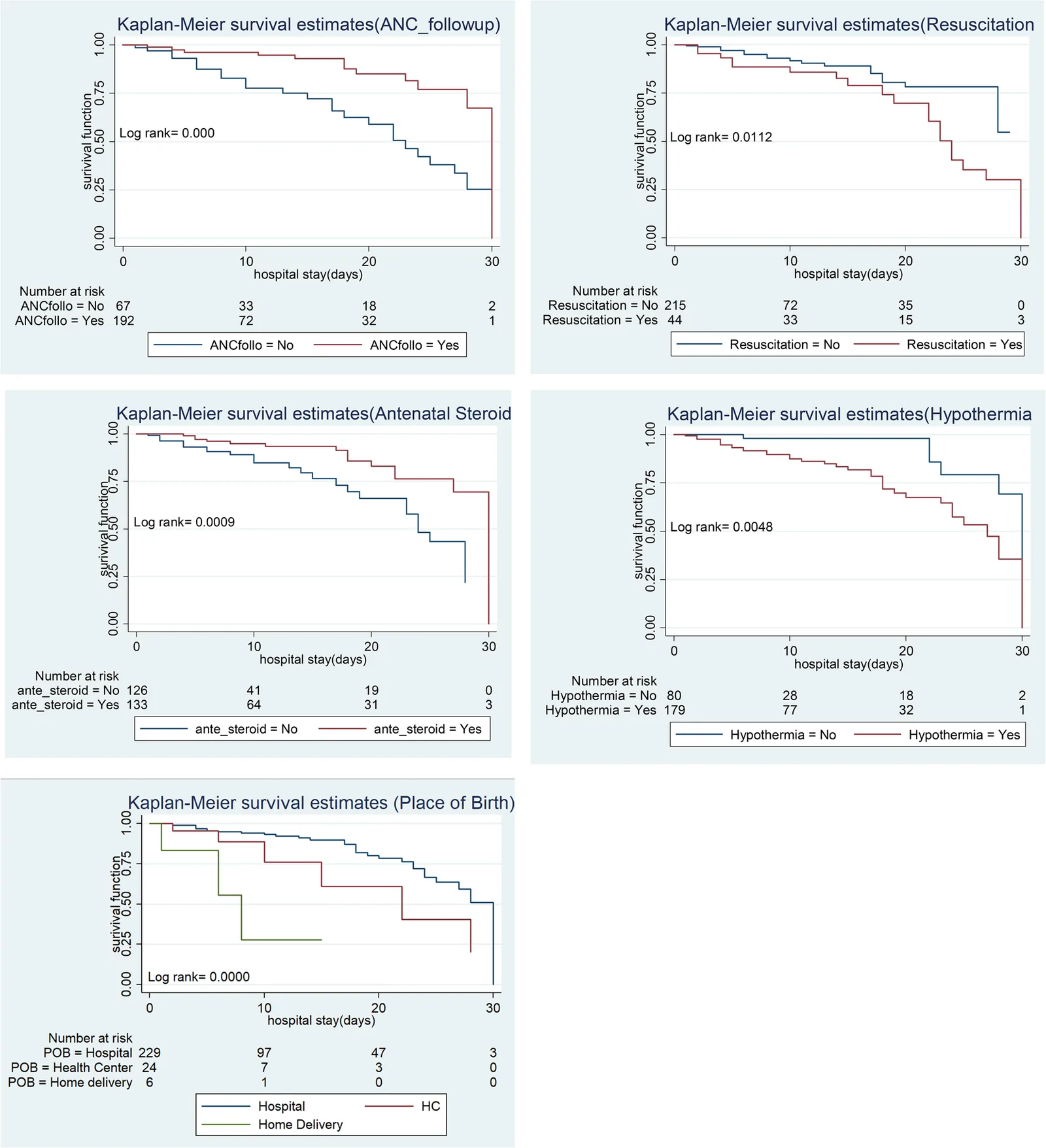
Kaplan–Meier Curve to compare survival probability by ANC follow-up, Antenatal steroid use, Place of birth, Resuscitation at birth, and Neonatal complication of preterm neonates at WUCSH

Separate Kaplan–Meier graphs were constructed to compare the survival probability across different covariates. In the Kaplan–Meier survival curves, one curve lying above the other indicates that the group represented by the higher curve has a longer survival time compared to the group with the lower curve. The log-rank test was used to assess the statistical significance of differences in survival probability among the groups at a 5% significance level (Figs. 5).

## Discussion

This Study is aimed to assess incidence and predictors of mortality among preterm neonates admitted to the NICU of Wallaga University Comprehensive Specialized Hospital.

The incidence proportion of preterm mortality observed in this study is lower than rates reported in other settings. Studies from Tikur Anbessa, Gondar, and Mizan Tepi reported higher proportions ranging from 28.8% to 35% [18, 20, 21], while a study in Southern Ethiopia reported 25.5% [17]. Outside Ethiopia, mortality rates were 31.6% in Uganda [22], 20.7% in Sierra Leone [23], and 23% in the India-Pakistan PURPOSe cohort [24]. These differences may reflect variations in sample size, follow-up duration, and population characteristics.

Studies from Ethiopia have reported shorter median survival times and higher incidence rates than this study. Tikur Anbessa Specialized Hospital found a median survival of 21 days with an incidence rate of 39.1 per 1,000 neonate-days [18]. Mizan Tepi reported 15 days and 62.15, respectively [21], while a study from Southern Ethiopia showed 18 days with a rate of 47.7 [17]. At Jimma University Medical Center, the incidence rate was 28.9 per 1,000 neonate-days [25]. These variations may reflect differences in study design, population characteristics, or NICU care quality.

Lack of ANC follow-up was significantly associated with increased mortality among preterm neonates. This finding is consistent with studies conducted in Southern Ethiopia, Mizan Tepi, and Uganda [17, 21, 22]. The possible explanation could be that mothers without ANC follow-up may miss opportunities for early detection and management of pregnancy related complications, counseling on danger signs, and timely referral to hospital and neonatal care. Inadequate ANC follow-up may therefore increase the likelihood of adverse perinatal outcomes and neonatal complications that contribute to mortality.

Use of antenatal steroid significantly reduced the risk of mortality among preterm neonates. This finding is supported by studies from Jimma and global meta-analyses [25, 26]. The protective effect may be explained by the role of antenatal steroid in accelerating fetal lung maturation, reducing RDS, IVH, and other complications related to prematurity.

Home delivery was strongly associated with preterm neonatal mortality. Similar findings were reported from Gondar and other studies conducted in low resource settings [20]. The increased risk of mortality among home delivered preterm neonates may be related to delayed initiation of essential newborn care, absence of skilled birth attendants, poor thermal care, delayed referral, and limited access to neonatal resuscitation services. The AHR for home delivery showed a wide 95% confidence interval, which is related to the small sample size within this subgroup. This wide interval indicates reduced precision of the estimate and suggests that the magnitude of association should be interpreted cautiously. However, the association remained statistically significant and was consistent with previous studies.

Neonatal hypothermia was also identified as an important predictor of mortality, which is consistent with findings from Bench Sheko and Sierra Leone [23, 27]. This may be explained by the physiological vulnerability of preterm neonates to heat loss due to immature thermoregulation, low brown fat stores, and large body surface area to body ratio. In addition, hypothermia may worsen respiratory distress, metabolic instability, and susceptibility to infection, thereby increasing the risk of neonatal death.

## Conclusion

The incidence proportion and incidence rate of preterm neonatal mortality in this study were lower compared with findings from previous studies. However, several important predictors of mortality were identified including lack of ANC follow-up, home delivery, and neonatal hypothermia, while antenatal steroid use was found to reduce the risk of death. These findings highlight the importance of improving antenatal care utilization, promoting institutional delivery, strengthening thermal care, and ensuring timely administration of antenatal corticosteroids to improve survival among preterm neonates.

## Supplementary Information

Supplementary Material 1.

## Data Availability

The datasets used during the current study are not publicly available due to patient confidentiality and institutional policies but are available from the corresponding author on reasonable request with permission from the institution.

## Abbreviations

ANC: Antenatal Care
AHR: Adjusted Hazard Ratio
APH: Antepartum Hemorrhage
BMI: Body Mass Index
BPD: Bronchopulmonary Dysplasia
EONS: Early Onset Neonatal Sepsis
GA: Gestational Age
GDM: Gestational diabetes mellitus
HMIS: Health Management Information System
IQR: Interquartile Range
IVH: Intraventricular Hemorrhage
KMC: Kangaroo Mother Care
NEC: Necrotizing Enterocolitis
NICU: Neonatal Intensive Care Unit
PROM: Premature Rupture of Membranes
RDS: Respiratory Distress Syndrome
WHO: World Health Organization
WUCSH: Wallaga University Comprehensive Specialized Hospital
