# Supplementary material for: Incidence and predictors of mortality among preterm neonates admitted to the neonatal intensive care unit of Wallaga University Comprehensive Specialized Hospital, Nekemte Town, East Wallaga, Oromia, Ethiopia: a retrospective cohort study
Source: BMC Pediatr. 2026 Jun 3;26:730. doi: 10.1186/s12887-026-07059-z (PMC13459156; doi:10.1186/s12887-026-07059-z)
Supplement: Supplementary file 1 [file 12887_2026_7059_MOESM1_ESM.docx]

# ANNEX I: MULTI COLLINARITY TEST

To ensure the reliability of the multivariable Cox regression model, multi-collinearity test was performed among the independent variables. The Variance Inflation Factor (VIF) was calculated for each predictor, and all values were found to be below 2. This indicates that there was no significant correlation between independent variables that could negatively influence the model's estimates. Therefore, the assumption of multi-collinearity was not violated, confirming that the independent variables included in the final model were appropriate and did not influence each other.

Multi-collinearity check for each variable based on Bivariate outcome (p <0.2).

| **Variable** | **VIF** | **1/VIF** |
| --- | --- | --- |
| ANC Follow up | 1.03 | 0.96 |
| Antenatal Steroid use | 1.01 | 0.99 |
| Place of birth | 1.03 | 0.96 |
| Resuscitation at birth | 1.08 | 0.92 |
| Hypothermia | 1.02 | 0.98 |
| Anemia | 1.06 | 0.94 |
| IVH | 1.04 | 0.96 |
| Seizure | 1.12 | 0.89 |
| **Mean VIF** | **1.05** |  |

# ANNEX II: PROPORTIONAL HAZARD ASSUMPTION

The proportional hazards assumption was checked using both graphs and statistical methods, and it was found to be met. The log-log plots showed that the lines were roughly parallel, which means the assumption was not violated. Also, the Schoenfeld Residuals test was checked and the global test was performed to test the hypothesis that the hazard ratios are constant over time. The global test was statistically insignificant (P= 0.0659) greater than the tabulated level of significance (0.05). This means it was appropriate to use the Cox regression model in this study.

# ANNEX III: MODEL GOODNESS-OF-FITNESS

The overall goodness-of-fit of the Cox proportional hazards model in this study was assessed using the Cox-Snell residual plot. The plot showed that the residuals closely followed the 45-degree reference line, indicating that the model fits the data well. This alignment suggests that the estimated survival function adequately represents the observed survival experience of the study population. Therefore, the Cox model was deemed appropriate for analyzing the factors associated with the survival of preterm neonates in this study.

**Figure 6: Cox Snell residual test to test the overall adequacy of the model.**
